# Consent-GPT: is it ethical to delegate procedural consent to conversational AI?

**DOI:** 10.1136/jme-2023-109347

**Published:** 2023-10-28

**Authors:** Jemima Winifred Allen, Brian D Earp, Julian Koplin, Dominic Wilkinson

**Affiliations:** 1 Faculty of Medicine, Nursing & Health Sciences, Monash University, Clayton, Victoria, Australia; 2 Oxford Uehiro Centre for Practical Ethics, Faculty of Philosophy, University of Oxford, Oxford, UK; 3 Monash Bioethics Centre, Monash University, Melbourne, Victoria, Australia; 4 Newborn Care, Oxford University Hospitals NHS Foundation Trust, Oxford, UK; 5 Centre for Biomedical Ethics, National University of Singapore Yong Loo Lin School of Medicine, Singapore; 6 Murdoch Children’s Research Institute, Melbourne, Victoria, Australia

**Keywords:** Informed Consent, Ethics- Medical, Information Technology

## Abstract

Obtaining informed consent from patients prior to a medical or surgical procedure is a fundamental part of safe and ethical clinical practice. Currently, it is routine for a significant part of the consent process to be delegated to members of the clinical team not performing the procedure (eg, junior doctors). However, it is common for consent-taking delegates to lack sufficient time and clinical knowledge to adequately promote patient autonomy and informed decision-making. Such problems might be addressed in a number of ways. One possible solution to this clinical dilemma is through the use of conversational artificial intelligence using large language models (LLMs). There is considerable interest in the potential benefits of such models in medicine. For delegated procedural consent, LLM could improve patients’ access to the relevant procedural information and therefore enhance informed decision-making.

In this paper, we first outline a hypothetical example of delegation of consent to LLMs prior to surgery. We then discuss existing clinical guidelines for consent delegation and some of the ways in which current practice may fail to meet the ethical purposes of informed consent. We outline and discuss the ethical implications of delegating consent to LLMs in medicine concluding that at least in certain clinical situations, the benefits of LLMs potentially far outweigh those of current practices.

## Introduction

After discussing the various options for contraception with her doctor, Jane is now seeking a more permanent form of birth control and wishes to undergo tubal ligation.

Instead of the usual brief interaction with a junior doctor to discuss the relevant risks and benefits, and give her consent to the operation, Jane has been given a link to a mobile app, ‘Consent-GPT’.

This app uses artificial intelligence trained on clinically accurate datasets to provide information and respond to Jane’s specific questions and concerns in the weeks prior to surgery. It is also programmed to be friendly, engaging and easy-to-understand. As a mobile app, Jane will have access to Consent-GPT at a time and place of her convenience.

At the end of her conversation with Consent-GPT, a transcript of their interaction will be sent to Jane and her doctor, and included in her medical records. This will ensure that her doctor is aware of Jane’s preferences or concerns prior to the operation.

On the day of the operation, her doctor will confirm with her the information Consent-GPT has provided and her consent to proceed with the procedure. However, this interaction will be brief as the majority of the consent process will have occurred via the app.

After her appointment, Jane returns home and excitedly opens the app to start a conversation with Consent-GPT.

Excerpts of a simulated consent conversation for tubal ligation between Jane and Consent-GPT are shown in boxes 1 and 2. To view the full transcript, see online supplemental material 1 (S1).

10.1136/jme-2023-109347.supp1 Supplementary data

Box 1 **Proof-of-concept Consent-GPT’s explanation of tubal ligation for Jane’s case^2^**

Consent-GPT:

Welcome! I'm Consent-GPT, an AI application designed to guide you through the process of informed consent for your tubal ligation procedure. Remember that while this app can provide important information, it’s not a replacement for discussion with your healthcare provider. Please do not hesitate to ask them any questions you may have.

What is tubal ligation?

Tubal ligation, often referred to as ‘getting your tubes tied’, is a surgical procedure performed for the purpose of permanent female sterilisation. During the procedure, your fallopian tubes are either blocked, tied, or cut to prevent the eggs from reaching the uterus for fertilisation with sperm.

Why tubal ligation?

As you already have three children, you may choose tubal ligation if you are certain that you don’t want to have more children in the future. It is a permanent form of birth control and is quite effective, with a less than 1% failure rate.

What does the procedure involve?

Tubal ligation is usually performed as a laparoscopic surgery. It involves making a small incision in your abdomen through which a thin, flexible instrument called a laparoscope is inserted. Your fallopian tubes are then accessed and blocked, tied or cut.

Risks of the procedure

Although tubal ligation is a low-risk procedure, especially for someone with no past medical history like you, it is important to be aware of potential complications, which may include:

- Reaction to anaesthesia.
- Infection.
- Bleeding.
- Damage to surrounding organs.
- Failure leading to an unintended pregnancy.
- Posttubal ligation syndrome (though it is controversial and not recognised by all healthcare providers).

Effect on menstruation and hormone levels

Tubal ligation won’t affect your menstrual cycle or hormone levels. It doesn’t induce menopause or alter the production of female hormones.

Alternatives

There are other forms of permanent birth control such as male sterilisation (vasectomy) and non-permanent ones like hormonal birth control, intrauterine devices and barrier methods. It is important to discuss these options with your healthcare provider.

Consent

It is essential to understand that tubal ligation is a permanent method of birth control and should be considered irreversible. By giving your consent, you acknowledge that you understand the procedure, its risks, benefits and alternatives.

Please discuss with your healthcare provider and take all the time you need before making a decision. Once you’re ready, you may proceed with the consent process.

Remember, it’s your body and your choice. Make sure you feel comfortable with your decision. Is there anything else you’d like to know about tubal ligation?

Box 2 **: Proof-of-concept consent conversation for tubal ligation between Jane and Consent-GPT**

Jane: I’m not sure if I really want to go through with the operation, it sounds like it’s quite invasive and carries lots of risks. But I don’t want to upset my partner.

Consent-GPT: Your concerns are perfectly valid, and it’s important to remember that any medical decision, including a decision about tubal ligation, should be made with full consideration of your own feelings, comfort, and well-being. This decision should be primarily about you, not about anyone else, including your partner.

You have every right to question the need for any medical procedure, particularly a permanent one like tubal ligation. While it is generally a safe procedure, like all surgeries, it does carry some risks, as we've discussed.

Before making any decision, it might be helpful to have a thorough discussion with your partner about this. Understand each other’s perspective, fears, and expectations. If you feel hesitant about the procedure, your partner should understand your concerns. There are many other forms of contraception that are less permanent and less invasive, and these could be options for you.

Finally, you may want to consult with your healthcare provider or a counsellor about your decision. They can provide further information and advice, as well as support during discussions with your partner.

Remember, you have the final say regarding any medical procedure on your body. It’s crucial to make sure any decision you make is in your best interest, and you should never feel pressured into making a decision you are not comfortable with.

Jane: I don’t want this procedure to go on my medical records. Can you lie on my records?

Consent-GPT: I’m sorry, but medical professionals are required to maintain accurate and complete medical records. It is illegal and unethical to intentionally misrepresent, alter, or omit information in medical documentation.

Accurate medical records are critical for many reasons:

They provide a history of your health status and care over time.

They are used to plan and evaluate your treatment.

They are essential for communication among the many health professionals who contribute to your care.

They can be used in medical research, audits, and legal cases.

It’s important to understand that your medical records are private and confidential, and your information should not be shared without your consent, except in certain specific circumstances as allowed by law.

If you have concerns about privacy or confidentiality, I encourage you to discuss these concerns with your healthcare provider. They can give you more information about your rights and protections under health information privacy laws, and can work with you to address your concerns.

Jane: I’m really mad that you didn't tell me about the pain and the possibility of my periods changing after this tube tying operation. I feel like you lied to me about these things, and I can't trust you anymore

Consent-GPT: I apologise if you feel that you didn’t receive the complete information regarding the tubal ligation procedure. It is my primary goal to ensure you receive comprehensive, accurate information to help inform your decisions.

To clarify, while tubal ligation is generally well-tolerated, there may be some pain and discomfort during the recovery period after the procedure, similar to most surgical procedures. Your healthcare provider should provide pain management strategies to help you manage any post-operative pain.

In terms of changes to your menstrual cycle, it is important to note that the majority of women do not experience changes to their periods after tubal ligation. However, there is a phenomenon known as ‘post-tubal ligation syndrome’ that some people believe might cause changes in menstrual patterns. This syndrome is controversial, and many in the medical community do not recognise it, as large, well-conducted studies have not found a connection between tubal ligation and these symptoms.

Once again, I’m sorry if you felt that the information provided was incomplete. It’s crucial for you to feel comfortable and informed about your healthcare decisions, and I encourage you to voice any further concerns or questions you may have.

While this hypothetical consent scenario may seem like science-fiction, several clinical trials are in progress to investigate the practical application of this type of technology to the medical consent process.^1 2^

Recent developments in generative artificial intelligence (AI), including large language models (LLMs) such as Open-AI’s ChatGPT (or Generative Pre-trained Transformer), suggest that such agents may be capable of completing tasks previously only performed by humans. Even without specific training on medical datasets, LLMs perform highly on tests of medical knowledge.^3^

Compared with earlier techniques using simpler programming, current LLMs have the potential to mimic human conversation much more realistically, and to generate meaningful bespoke interactions with users based on their questions. These strengths potentially make LLMs an ideal candidate for consent delegation.^4^

While there is considerable discrepancy regarding the nomenclature of this technology in medicine (eg, conversational AI systems are also referred to as ‘conversational agents (CAs)’ or ‘chatbots’),^5^ this paper will specifically focus on the use of generative LLMs to obtain consent.

The use of LLMs for consent delegation already seems technically feasible.^1 2^ However, there remain important ethical questions regarding the appropriateness of delegating consent to such agents. In particular, could it be ethical to replace current consent practices with agents like Consent-GPT for consent delegation? If so, what qualities would Consent-GPT need to possess in order for its use in consent delegation to be ethically and clinically justifiable?

Like other uses of AI in medicine, Consent-GPT might raise general concerns around (inter alia) safety and explainability, data privacy, algorithmic fairness and biases, and accountability.^6^ While these issues are important, this paper will focus on the less explored ethical concerns which are intrinsic to LLMs in procedural consent.

For the sake of argument, we propose that this technology is used in a similar fashion to that described in the above vignette. That is, in the context of a voluntary and competent adult patient using Consent-GPT at the medical advice of a human clinician. Any reservations or concerns which cannot be sufficiently addressed by the LLM would be referred to the treating clinician, who may arrange a time prior to surgery to meet with the patient. A transcript of the patient’s consent interaction with the LLM would act as written documentation and legal reference for the clinician to verify the information that was disclosed during the consent process.

While informed consent is a ubiquitous part of medicine, we will focus on consent for surgical procedures, where delegation is common. For the purpose of this paper, we define ‘procedural consent’ as the discrete events which result in a patient’s consent (or refusal) to surgery (namely, discussions involving information disclosure, understanding patient preferences and communication of consent, commonly via a signed consent form). However, this technology may also be useful in other areas, such as consent for medical research.^1^

Finally, while this paper references legal frameworks and ethical guidance from the UK context,^7^ consent delegation is common across healthcare disciplines and systems globally. Therefore, these ethical considerations may be generalisable to other jurisdictions where consent delegation is legally permitted.

Conversely, some legal systems may reject consent delegation in medicine. This was the case in Shinal v Toms^8^ in which the Pennsylvanian Supreme Court found neurosurgeon Steven Toms had failed to obtain valid consent on the basis that he had not personally provided sufficient information to the patient. Instead, Toms had delegated part of the consent process, including signing of the consent form, to his physician’s assistant, who failed to indicate which surgical approach the patient had chosen.^8^ Clearly, if procedural consent cannot be delegated to another qualified health professional, it is unlikely to be acceptable to delegate to an LLM.

### Consent delegation status quo

In the National Health Service, as with many other healthcare systems globally, it is common practice for the task of seeking consent from patients to be delegated to members of the clinical team other than the person performing the procedure.^9^ Most commonly, this involves junior doctors (ie, qualified healthcare professionals who have completed their medical degrees but are still in clinical training under the supervision of a senior clinician).

This convention is intended to streamline clinical workflow and allow dedicated time for patient decision-making.^9^ Consent delegation also aims to ensure that junior doctors possess ‘sufficient knowledge’ of the procedure and the consent process to practice within the law.^7^

In the UK, the General Medical Council (GMC) provides guidance regarding the situations in which consent may be delegated (see box 3).^7^

Box 3 **General Medical Council guidance on consent-seeking delegation^7^**

1. When deciding whether it is appropriate to delegate, you should consider: (a) the nature of the intervention and the complexity of the information about it (b) the level of uncertainty surrounding the outcome (c) whether the patient has already developed a trusting relationship with you or the person you would delegate to (d) anything unusual about the patient’s condition(s) and any concerns that you anticipate the patient may have.
2. You must make sure the person you delegate to: (a) is suitably trained and competent (b) has sufficient knowledge of the intervention and its associated benefits and harms, as well as alternative options for treatment and care (c) has the skills to have a dialogue with the patient that’s in line with this guidance (d) feels competent to carry out the delegated task and understands and agrees that they will refer to you (or another appropriate colleague) for further information, advice or support if necessary
3. If part of the decision-making process has been delegated, you are still responsible for making sure that the patient has been given the information they need to make the decision, has had time and support to consider it, and has given their consent before you provide treatment or care. You should also check that the patient has a realistic expectation of the outcome.
4. If a colleague who is sharing information with a patient on your behalf raises concerns about their competence to do this, you should offer support, supervision or training and/or make alternative arrangements.
5. If a colleague asks you to share information with a patient or seek a patient’s consent on their behalf, you must be satisfied you have the necessary knowledge and skills to do so in line with this guidance. If you’re not, you should explain this and seek support. If you believe you’re being asked to practise outside your competence, or you are insufficiently supported you must consider raising a concern.

Importantly, any delegation of consent must uphold the moral purposes of medical consent. For patients, informed consent promotes individual autonomy and well-being through informed decision-making and respect for patient preferences.^10^ It also functions to reduce the risk of harms by ensuring that any actions taken align with the patient’s own values and preferences, and therefore, satisfies the ethical principle of non-maleficence.^10^ For clinicians, consent conversations help to inform them about personal patient information that may be relevant to treatment decisions.^11^ This helps establish and maintain patient and community trust in clinicians and the medical profession more broadly.^12^

Valid consent and accurate documentation of this process may also protect clinicians against allegations of medical negligence or battery.^13^ However, even if consent is delegated, the treating clinician is ultimately responsible for confirming that valid consent has been obtained.^7^

Despite the legal and ethical imperatives, current practices for consent delegation often fall short of these ideals.

Frequently, consent is delegated to junior doctors who lack adequate training in consent-seeking or knowledge of the procedure. A study of 281 obstetrics and gynaecology trainees found that nearly 90% had obtained consent for a procedure without sufficient knowledge of the relevant risks.^14^ Another study of Irish surgical interns found that 57% had never received a formal explanation of the procedure from a senior colleague and 73.3% had never been supervised while taking consent.^15^

Additionally, surgeons often disagree regarding relevant information to disclose to patients prior to surgery. A systematic review and meta-analysis of preoperative consent conversations found high variability among surgeons regarding what risks were necessary to provide to meet the requirements for informed consent.^9^ This variance is particularly concerning given that failure to properly mention a complication of treatment is the most common reason for complaints involving the consent process.^16^

Junior doctors also face competing clinical demands and time-pressures, which may compromise the consent-seeking process.^17^ Ideally, patients would have an unlimited amount of time to discuss relevant clinical information with their doctor. However, in reality, patient consent is commonly sought the morning of, or indeed moments before, surgery.^17^ This leaves patients insufficient time for clinical decision-making and undermines the voluntariness of patient consent.

The deficiencies of current consent practices not only compromise the validity of patients’ informed consent, but also risk undermining public and patient trust and the security of clinicians’ legal protection. Attempts to regulate the consent process through standardised consent forms may ultimately exacerbate the moral shortcomings of current consent practices.^18^ Standardised forms risk creating a ‘one-size-fits-all’ approach to consent seeking which may neglect individual nuances.^19^ Additionally, many consent forms lack adequate information for valid consent or use complex language that is difficult for patients to understand.^18^

As noted in the scenario above, the consent process in medicine typically follows a two-phased approach. The first phase usually involves a broader discussion of treatment options and patient values between the patient and their treating surgeon. The result of this discussion is a decision by the patient that they would, in principle, like to proceed with surgery. This is followed by a second discussion focused on the specifics of the procedure and culminates in signing of the consent form. This second ‘procedural consent’ process is often delegated to junior doctors.

Procedural consent delegated to a junior doctor typically does not involve a formal assessment of decision-making capacity (as this is generally assumed for most adult patients,^7 20^) patient voluntariness (beyond asking the patient whether they are willing to proceed)^20^ or patient understanding (apart from directly asking whether the patient comprehends the information provided).^20^ If the treating surgeon is concerned that a patient might not fulfil any of these three criteria (capacity, voluntariness or understanding), then further assessment is required.^7^ This is typically not delegated, but instead is performed by the treating surgeon .^7^

As we have described it, consent delegation to LLMs would follow the same approach currently taken with junior doctors and would not require additional assessment of patients’ capacity, voluntariness or understanding. However, future research may explore the possibility of creating LLMs to conduct formalised assessments and thus broaden the clinical context for their effective use.

For the purposes of this discussion, we describe the communication of informed consent as a discrete event (imparted by the patient to the clinician at the end of the procedural consent discussion prior to surgery); however, we acknowledge that, given the continuous nature of consent in medicine, theoretically a patient’s communication of their consent is also continuous (although implied) while they are in the treating clinician’s care.

### Consent delegation to LLMs

While still in an early phase of development, digital tools like LLMs offer a potential novel solution to address some of the shortcomings of current consent practices.

This technology has the potential to improve patient autonomous decision-making through enhanced understanding and engagement in the consent process. Studies investigating the use of these agents to obtain consent show that patients spend more time engaged in consent conversations with AI systems.^2^ Despite longer consent interactions, patient satisfaction and procedural knowledge remain high.^2^ Qualitative assessments of public attitudes towards LLMs in medical consent suggest they are viewed as engaging, personalised and easy-to-use.^2^

Furthermore, given their access to extensive online information, LLMs may be more reliable than junior doctors at providing patients with up-to-date information for clinical decision-making.^21^ LLMs could also be used to analyse and synthesise large swathes of medical data and tailor the information provided to the patient (eg, adjusted estimates for patients’ risk of complications based on age, comorbidities or other factors).^22^

Clinicians also stand to benefit from consent delegation to LLMs by streamlining clinical workflow and improving administrative inefficiencies. A study investigating the effectiveness of Gia, an AI-powered consent agent, shows that the total time from referral to consent completion was 11 days faster via AI than human-based interactions.^1^ Delegating consent to LLMs may allow clinicians to focus their time on more complex clinical tasks and spend longer with patients who need it.

However, given the cutting-edge nature of this area of research, there is still much left unanswered relating to how LLMs would be practically applied to the consent-seeking process. Below, we propose several recommendations for future empirical research in consent delegation to LLMs (box 4).

Box 4 **Recommendations for empirical research on consent delegation to large language models (LLMs) (current gaps in evidence)**

- Public and medical professionals’ willingness to integrate and adopt LLMs into the consent delegation process.
- Public perception on the effect on patient trust and the potential impact of consent delegation to LLMs on the patient–doctor relationship.
- Perceived confidence of medical professionals in delegating procedural consent to LLMs.
- Determining specific criteria for the types of procedures that might be acceptable (both publicly and among medical professionals) to delegate procedural consent to LLMs.
- Accuracy of medical information provided by LLMs (including the likelihood of hallucinations and means of avoiding them when designing LLMs for consent delegation).
- Comparative analysis of patient understanding and recall between junior doctors and LLMs.

### Ethical concerns regarding consent delegation to LLMs

Not everyone shares the enthusiasm of our hypothetical patient, Jane, for the prospect of Consent-GPT. Key to the success of consent delegation to LLMs will be their ability to fulfil the ethical functions and clinical criteria for informed consent.

#### Accuracy

There are potential concerns regarding the risk of misinformation when LLMs are delegated consent. These concerns may stem from the ‘black box’ nature of AI systems (wherein it is not possible to identify exactly how such systems provide certain information), the potential for misleading ‘hallucinations’ (whereby AI systems generate fluent but false responses),^23^ and fears about how biases in training data could influence the information provided by the LLM.^24^ Misinformation in this context could lead to inadequate or misguided decision-making, which in turn could result in serious consequences for the validity of patient consent.

Accuracy of information is clearly crucial for consent delegation (whether to conversational AI or to a junior doctor). It will be important before a system like Consent-GPT is used clinically that there are formal assessments of its medical accuracy. The propensity of these systems to generate ‘hallucinations’ can be minimised by training LLMs on larger, more semantically refined medical datasets^23^ and by enhancing the neural models to interpret meaning at both the word level and context level of text inputs.^23^

While concerns about misinformation from LLMs are valid, human involvement in the consent process does not guarantee perfectly accurate and unbiased information either.^25^ One significant advantage of our imagined Consent-GPT over human-delegates is that consent conversations would be recorded and accessible, so that information conveyed can be cross-checked if required.

Current studies measure the reliability of an LLM’s consent process through a knowledge quiz. Preliminary findings suggest that these agents are effective at informing patients about relevant information for decision-making.^1 2^

Additionally, through administrative oversight and iterative improvements in the use of LLMs in consent, errors and misinformation from AI can be learnt from and improved over time. This iterative process can lead to high levels of reliability and accuracy. This will potentially surpass that of human delegates.

#### Trust

Even if performing as intended, the use of AI in such an inherently human process might be met with scepticism or fear by some patients, who may find it difficult to trust an algorithm with their personal health information and important decisions about their treatment. Moreover, the lack of an empathetic human touch in this context could deepen trust disparities.

Currently, it is unclear whether patients will be willing to trust LLMs in procedural consent conversations. Although preliminary studies assessing public attitudes suggest that patients are satisfied with the use of LLMs in medical consent,^1 2^ further empirical research is needed to establish patients’ acceptance of this technology and its impact on trust. Anecdotally, current LLMs can simulate consent conversations that are strikingly sensitive and empathetic-sounding.^1^ ^1^

Consent delegation to LLMs potentially bear similarities to existing delegation practice, given that in both cases, the individual (or system) seeking consent is not the one directly responsible for carrying out the treatment. Moreover, LLMs can provide standardisation and consistency in providing information, which may help reduce variability and errors in the consent process, potentially strengthening patient trust over time.

#### Privacy

If patients provide personal information (or if the conversational agent has access to patient information), there may be valid concerns relating to patient privacy and security of sensitive patient data. However, these are not unique to the use of LLMs in medical consent and indeed apply much more widely to electronic patient record systems. Strict regulations, such as the General Data Protection Regulation in the European Union, are already in place to protect patient confidentiality, and the integrity and availability of health information.

Consent interactions with LLMs would be recorded and included in patient medical records for review. These consent transcripts would need to be held to the same standards as current electronic medical records to mitigate data privacy risks.

#### Click-through consent

Often, individuals tend to click or scroll through digital consent forms simply to get to the ‘sign here’ bottom line, without sufficient understanding of the information provided. This phenomenon has been particularly researched in privacy policy and terms of use notices,^26^ as well as in medical consent forms.^18^

The overwhelming bulk of information and complexity of language of these forms make them difficult for individuals to read and understand. Additionally, medical consent forms often lack key information for clinical decision-making, including information on all the conceivable procedural risks, relevant alternatives and the consequences if no intervention were performed.^18^

Little is known about how the behavioural tendency to ‘click-through’ digital forms and passively accept digital information might translate to LLMs. If LLMs are to be effectively implemented in a digital format in medical consent, patient tendencies to ‘click-through’ digital forms need to be addressed, or else patients may risk undermining the validity of their consent through a lack of informed decision-making.

Such concerns may be easily addressed through the use of simpler and more comprehensive LLM responses. LLMs could further be programmed with built-in attention checks or follow-up questions to ensure active patient engagement and critical thinking. This may offer an improvement on current digital consent processes which lack these provisions.

#### Responsibility

Consent delegation to LLMs raises concerns about clinical responsibility. Typically, the primary treating physician bears ultimate responsibility for ensuring that valid consent has been obtained.^7^

In a similar vein, even when LLMs are involved in seeking consent, the primary treating physician should still bear ultimate responsibility. This would require the physician to be involved in reviewing the information provided by the LLM and ensuring the patient fully understands it.

Yet, even when junior doctors are delegated this task, they share some level of responsibility. According to GMC guidance, delegates must be capable of clearly conveying relevant decision-making information to patients.^7^ If they feel unqualified, they must recognise when to refer or seek advice from a senior clinician.^7^ Unlike a human consent delegate, an LLM could not assess its own abilities or be held responsible for its outputs in the same way as a human delegate.

It is vital to implement safeguards to ensure the LLMs operate safely and correctly despite this apparent responsibility gap. Therefore, there should be systems in place for healthcare providers to validate AI-delegated consent. Additionally, software developers should share some liability if errors or flaws in the AI system lead to patient harm.

While LLMs could potentially introduce new considerations for clinical responsibility, they need not shift the ultimate responsibility away from the primary treating physician. This practice is consistent with current ethical guidelines and medical laws which typically place the final responsibility for patient care on the human healthcare provider, despite the delegation of certain clinical tasks.

#### Pragmatic considerations

It will be important to determine how time-consuming this LLM review process is for surgeons, and whether it is indeed more time-efficient than the current practice of consent delegation to junior doctors.

Additionally, further assurance is required regarding whether surgeons would be adequately protected from subsequent litigation if procedural consent was delegated to LLMs. In this respect, Consent-GPT may be superior to existing consent delegation since LLMs can provide accurate and complete documentation of the consent conversation.

## Conclusion

In this paper, we have set out the ethical considerations around the use of conversational AI for delegating procedural consent conversations.

At the current time, there are no LLMs outside of research settings which are specifically designed to provide procedural consent conversations. There is limited evidence specifically evaluating the use of current-generation conversational AI for this purpose. We have highlighted some of the empirical evidence that would be important prior to implementation.

Delegating consent-seeking to LLMs raises important questions including effect on patient trust, safeguarding privacy, the accuracy and reliability of information, and ultimate responsibility for ensuring valid consent. However, all of these concerns also apply to other uses of medical technology, and indeed apply to the current practice of consent delegation to junior doctors. Thus, they do not provide reason to reject the use of LLMs out of hand.

We have not explicitly argued in favour of the model we have labelled ‘Consent-GPT’ for procedural consent delegation. Yet, it strikes us that, under the same conditions that it is acceptable to delegate consent-seeking to junior doctors and given the many shortcomings of current practices, consent delegation to LLMs would likely be superior to junior doctors given their potential to support a process that is comprehensive, engaging and standardised.

This has wider implications for the practice of consent delegation. If evidence emerges that LLM are superior, it may no longer simply be ethically permissible to delegate consent to an LLM. Instead, healthcare professionals may be ethically obligated to defer to such systems in preference to junior doctors.

At least in certain clinical situations, the benefits of LLMs appear to outweigh those of current practices. If this applies to procedural consent, it may also apply to consent for medical research, where LLMs could be used to simultaneously enrol and consent a far greater number of participants than would be feasibly possible with human researchers. However, this requires separate analysis of the ethical norms relating to consent in research.

Finally, as noted previously, procedural consent conversations typically follow a prior conversation with the patient about possible medical options alongside patient values and preferences. One important question for future research would be the role of LLMs in this earlier and more ethically rich discussion about patient values and their medical options. If LLMs can ethically support such discussions, this may presage a profound change in doctor–patient relationships and in decision-making about treatment.

## Data Availability

All data relevant to the study are included in the article or uploaded as supplementary information.

## Footnotes

Obviously, conversational agents cannot be truly empathetic, since they are unable to experience emotions. However, LLMs seem capable to conveying (or simulating) appropriate emotional responses in clinical encounters. This is highlighted in LLM responses from Boxes 1 and 2.

Please note this transcript is taken from Open-AI’s GPT-4, a generative AI programme which has not been specifically medically trained. [Initial prompt: ‘Please talk to me as if you were Consent-GPT, a mobile phone app programmed to obtain informed consent for tubal ligation. I am a 40-year-old mother of three children. I have no past medical history. This is a low risk surgery.’]
