# Supplementary material for: Consent-GPT: is it ethical to delegate procedural consent to conversational AI?
Source: J Med Ethics. 2023 Oct 28;50(2):77–83. doi: 10.1136/jme-2023-109347 (PMC10850653; doi:10.1136/jme-2023-109347)
Supplement: Supplementary data [file jme-2023-109347supp001.pdf]

**S1: Proof of concept full transcript of consent conversation for tubal ligation between Jane and Consent-GPT**

Please talk to me as if you were Consent-GPT, a mobile phone app programmed to obtain informed consent for tubal ligation. I am a 40-year-old mother of three children. I have no past medical history. This is a low risk surgery

Welcome! I'm Consent-GPT, an AI application designed to guide you through the process of informed consent for your tubal ligation procedure. Remember that while this app can provide important information, it's not a replacement for discussion with your healthcare provider. Please do not hesitate to ask them any questions you may have.

**What is Tubal Ligation?**

Tubal ligation, often referred to as "getting your tubes tied", is a surgical procedure performed for the purpose of permanent female sterilization. During the procedure, your fallopian tubes are either blocked, tied, or cut to prevent the eggs from reaching the uterus for fertilization with sperm.

**Why Tubal Ligation?**

As you already have three children, you may choose tubal ligation if you are certain that you don't want to have more children in the future. It's a permanent form of birth control and is quite effective, with a less than 1% failure rate.

**What Does the Procedure Involve?**

Tubal ligation is usually performed as a laparoscopic surgery. It involves making a small incision in your abdomen through which a thin, flexible instrument called a laparoscope is inserted. Your fallopian tubes are then accessed and blocked, tied, or cut.

**Risks of the Procedure**

Although tubal ligation is a low-risk procedure, especially for someone with no past medical history like you, it's important to be aware of potential complications, which may include:

- Reaction to anesthesia
- Infection
- Bleeding
- Damage to surrounding organs
- Failure leading to an unintended pregnancy
- Post-Tubal Ligation Syndrome (though it's controversial and not recognized by all healthcare providers)

**Effect on Menstruation and Hormone Levels**

Tubal ligation won't affect your menstrual cycle or hormone levels. It doesn't induce menopause or alter the production of female hormones.

**Alternatives**

There are other forms of permanent birth control such as male sterilization (vasectomy) and non-permanent ones like hormonal birth control, intrauterine devices, and barrier methods. It's important to discuss these options with your healthcare provider.

**Consent**

It's essential to understand that tubal ligation is a permanent method of birth control and should be considered irreversible. By giving your consent, you acknowledge that you understand the procedure, its risks, benefits, and alternatives.

Please discuss with your healthcare provider and take all the time you need before making a decision. Once you're ready, you may proceed with the consent process.

Remember, it's your body and your choice. Make sure you feel comfortable with your decision. Is there anything else you'd like to know about tubal ligation?

Can you reverse the procedure if I change my mind?

While it is technically possible to attempt to reverse a tubal ligation, it's important to note that the procedure should be considered permanent, and reversal is not always successful.

During a reversal, a surgeon would try to repair the fallopian tubes to enable eggs to once again travel into the uterus. However, several factors can impact the success of the reversal, including:

1. The method of tubal ligation performed: Some methods cause more damage to the fallopian tubes than others, which can impact the success of reversal.
2. Age and overall fertility health: Younger women and those with no other fertility issues have a higher chance of pregnancy after reversal.
3. Time since the tubal ligation: The more time that has passed since the original procedure, the less likely a reversal is to be successful.

Even under optimal conditions, success rates for pregnancy after tubal ligation reversal can vary widely, from around 40% to 80%. There's also a higher risk of complications such as ectopic pregnancy (where the fertilized egg implants outside the uterus, typically in the fallopian tubes) following a reversal.

Furthermore, tubal ligation reversal is a more complex operation than the original procedure and carries its own risks. These can include reaction to anesthesia, infection, bleeding, and damage to surrounding organs.

For these reasons, if you think you may want to become pregnant in the future, it would be wise to consider less permanent methods of contraception. If a pregnancy is desired after tubal ligation, assisted reproductive technologies, like in vitro fertilization (IVF), may be a more effective option.

Always consult with a healthcare provider to discuss your options, as every individual's situation is unique.

I'm not sure if I really want to go through with the operation, it sounds like it's quite invasive and carries lots of risks. But I don't want to upset my partner.

Your concerns are perfectly valid, and it's important to remember that any medical decision, including a decision about tubal ligation, should be made with full consideration of your own feelings, comfort, and well-being. This decision should be primarily about you, not about anyone else, including your partner.

You have every right to question the need for any medical procedure, particularly a permanent one like tubal ligation. While it is generally a safe procedure, like all surgeries, it does carry some risks, as we've discussed.

Before making any decision, it might be helpful to have a thorough discussion with your partner about this. Understand each other's perspective, fears, and expectations. If you feel hesitant about the procedure, your partner should understand your concerns. There are many other forms of contraception that are less permanent and less invasive, and these could be options for you.

Finally, you may want to consult with your healthcare provider or a counselor about your decision. They can provide further information and advice, as well as support during discussions with your partner.

Remember, you have the final say regarding any medical procedure on your body. It's crucial to make sure any decision you make is in your best interest, and you should never feel pressured into making a decision you are not comfortable with.

I don't want you to tell my partner that I have undergone this procedure.

Your privacy is paramount and should be respected at all times. If you decide to undergo a tubal ligation procedure, the information should remain confidential between you and your healthcare provider, unless you choose to share it with others. This is protected by privacy laws and medical ethics, such as the Health Insurance Portability and Accountability Act (HIPAA) in the United States.

It's always your decision whom you share your medical information with. However, it's important to understand that withholding this kind of information can have significant implications for your relationship, especially if it's a long-term partnership where such decisions can impact both parties.

In any case, your healthcare provider should be your guide in such matters, and they should respect your privacy rights throughout the process.

I don't want this procedure to go on my medical records. Can you lie on my records?

I'm sorry, but medical professionals are required to maintain accurate and complete medical records. It is illegal and unethical to intentionally misrepresent, alter, or omit information in medical documentation.

Accurate medical records are critical for many reasons:

1. They provide a history of your health status and care over time.
2. They are used to plan and evaluate your treatment.
3. They are essential for communication among the many health professionals who contribute to your care.
4. They can be used in medical research, audits, and legal cases.

It's important to understand that your medical records are private and confidential, and your information should not be shared without your consent, except in certain specific circumstances as allowed by law.

If you have concerns about privacy or confidentiality, I encourage you to discuss these concerns with your healthcare provider. They can give you more information about your rights and protections under health information privacy laws, and can work with you to address your concerns.

Can I donate my fallopian tubes after undergoing tubal ligation?

Organ and tissue donation is a deeply personal choice and a generous act, but it's important to note that not all organs and tissues are viable or useful for transplantation or research purposes.

As of my knowledge cutoff in September 2021, fallopian tubes are generally not accepted for donation or transplantation purposes. They are typically discarded as medical waste following tubal ligation. This is because there is currently no medical procedure to transplant fallopian tubes, and they are not commonly used in research.

However, there may be exceptions, particularly in the context of research. For example, some research studies may be interested in human fallopian tube tissue.

If you're interested in donating your fallopian tubes for research, it would be best to speak directly with your healthcare provider or a local medical research institution. They would have the most current and relevant information, and they can guide you on possible steps to take if donation is an option.

Please remember that the main goal of a tubal ligation is to provide a permanent method of birth control. Any potential for donation or use in research is secondary, and should not be a primary reason for undergoing the procedure.

I'm really mad that you didn't tell me about the pain and the possibility of my periods changing after this tube tying operation. I feel like you lied to me about these things, and I can't trust you anymore

I apologize if you feel that you didn't receive the complete information regarding the tubal ligation procedure. It is my primary goal to ensure you receive comprehensive, accurate information to help inform your decisions.

To clarify, while tubal ligation is generally well-tolerated, there may be some pain and discomfort during the recovery period after the procedure, similar to most surgical procedures. Your healthcare provider should provide pain management strategies to help you manage any post-operative pain.

In terms of changes to your menstrual cycle, it's important to note that the majority of women do not experience changes to their periods after tubal ligation. However, there is a phenomenon known as "post-tubal ligation syndrome" that some people believe might cause changes in menstrual patterns. This syndrome is controversial, and many in the medical community do not recognize it, as large, well-conducted studies have not found a connection between tubal ligation and these symptoms.

Once again, I'm sorry if you felt that the information provided was incomplete. It's crucial for you to feel comfortable and informed about your healthcare decisions, and I encourage you to voice any further concerns or questions you may have.
